# Application of Nano-Titanium Dioxide in Food Antibacterial Packaging Materials

**DOI:** 10.3390/bioengineering12010019

**Published:** 2024-12-29

**Authors:** Jiarui Li, Dequan Zhang, Chengli Hou

**Affiliations:** Institute of Food Science and Technology, Chinese Academy of Agricultural Sciences/Key Laboratory of Agro-Products Quality & Safety in Harvest, Storage, Transportation, Management and Control, Ministry of Agriculture and Rural Affairs, Beijing 100193, China; jiaruili1128@163.com (J.L.); dequan_zhang0118@126.com (D.Z.)

**Keywords:** nano-titanium dioxide, food packaging, antimicrobial, reactive oxygen species, photocatalytic

## Abstract

Food waste and food safety issues caused by food spoilage have been brought into focus. The inhibition of food spoilage bacteria growth is the key to maintaining food quality and extending the shelf life of food. Photodynamic inactivation (PDI) is an efficient antibacterial strategy which provides a new idea for the antibacterial preservation of food. Nano-titanium dioxide (nano-TiO_2_) with PDI characteristics has attracted the interest of many researchers with its elevated efficiency, broad-spectrum antibacterial resistance, low cost, safety, and non-toxicity. Nano-TiO_2_ photodynamic antibacterial properties have been studied extensively and has a great application value in the field of food packaging. The antibacterial properties of nano-TiO_2_ are linked to its photocatalytic activity and are influenced by factors such as reactive oxygen species production, bacterial types, etc. Polymer-based nano-TiO_2_ packaging has been prepared using various methods and applied in various foods successfully. In this review, the latest research on photocatalytic and antibacterial mechanisms and factors of nano-TiO_2_ is discussed, and its applications in food antibacterial packaging are also explored comprehensively. Challenges and future perspectives for nano-TiO_2_-based food packaging applications have been proposed. This review aims to provide a whole comprehensive understanding of novel antibacterial packaging systems based on nano-TiO_2_.

## 1. Introduction

Food spoilage causes serious food waste and foodborne diseases. Around the world, 240 million tons of protein-rich foods, such as meat, chicken, and fish products, are wasted annually [1], causing substantial economic losses. Microbial contamination and the enzymes secreted by these organisms are the main causes of food spoilage [2] and an important factor in food waste [3]. Therefore, antibacterial properties are the key to food preservation. In recent decades, with the development of antimicrobial technology, the frontiers of antibacterial strategies have been moving forward, whose research scopes have been far and wide beyond the antibacterial agents. Photodynamic inactivation (PDI) has attracted significant interest, and driven substantial advances in non-thermal antibacterial platforms, and has become a promising antibacterial method for food preservation [4]. Unlike conventional antibacterial methods, photosensitizers with PDI properties do not kill the bacteria directly. In contrast, it is the reactive oxygen species (ROS) produced by photosensitizers upon light stimulation that exert an antimicrobial effect.

Nano-titanium dioxide (nano-TiO_2_) is a kind of metal oxide with three different crystal structures in nature (such as rutile, anatase, and brookite). Its unique semiconductor structure makes it produce ROS under ultraviolet (UV) light irradiation and can kill many kinds of Gram-negative and Gram-positive bacteria such as *Escherichia coli*, *Staphylococcus aureus*, and *Listeria* easily. Additionally, its high surface area and small particle size, with a particle size distribution ranging from 1 to 100 nanometres (nm), are advantageous for enhancing its antibacterial effects. Moreover, it was approved by the United States Food and Drug Administration (FDA) for use in food contact materials. Nano-TiO_2_ is a reliable photosensitizer with PDI characteristics. Due to its broad antimicrobial spectrum, low toxicity or non-toxicity, and high antimicrobial efficiency, it has sparked significant interest in the food packaging material industry as functional antibacterial nanoparticles.

Previous studies showed that nano-TiO_2_-based food packaging has excellent antibacterial properties under UV irradiation, which can reduce the initial number of microorganisms on the surface of packaged food and effectively extend the shelf life of food [5,6,7]. Moreover, the success of PDI of nano-TiO_2_-based food packaging significantly hinges on the careful selection of nano-TiO_2_ and polymer matrices for synthesizing polymer-based photoactive materials. Although several reviews have been published on the antimicrobial activity of nano-TiO_2_ and its applications in food antimicrobial packaging [8,9,10], these reviews primarily focused on the classification and synthesis method of nano-TiO_2_, and their impact on packaging performance. However, there is a lack of comprehensive theoretical guidance on the entire process, from selecting nano-TiO_2_ to fabricating polymer-based packaging materials, enhancing the photocatalytic antimicrobial activity of the systems, evaluating the antimicrobial properties, and, finally, applying nano-TiO_2_-based food packaging.

Therefore, this paper aims to elucidate the mechanism of photocatalytic antibacterial properties and key factors affecting the generation and utilization efficiency of ROS, providing a systematic theoretical reference for efficient antibacterial activity. Applications in food packaging are discussed, including nano-TiO_2_-based film preparation methods, antibacterial testing and evaluating methods, application progresses in food preservation, and the migration of nano-TiO_2_ from packaging into food. Finally, the prospects for future practical production applications of nano-TiO_2_ food antibacterial packaging are elaborated.

## 2. Photocatalytic and Antibacterial Performance of Nano-TiO_2_

**Figure 1:**
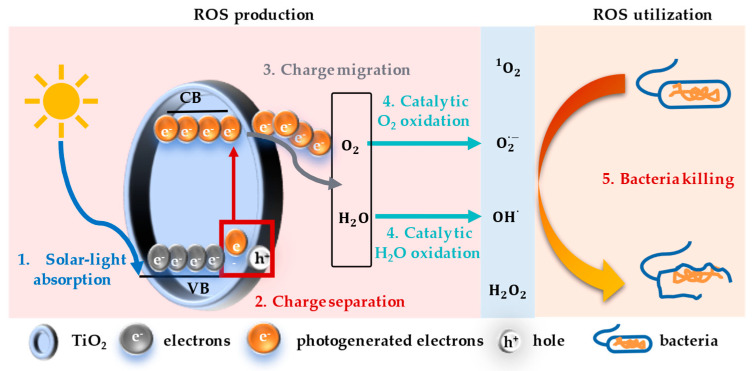
Photocatalytic and antibacterial properties of nano−TiO_2_. Reprinted (adapted) with permission from Ref. [11]. Copyright 2023 American Chemical.

Nano-TiO_2_ exhibits excellent photocatalytic and antibacterial properties. ROS production through photocatalysis and their utilization in the antibacterial process contribute to bacteria inactivation (Figure 1). To further enhance the photocatalytic antibacterial properties of nano-TiO_2_ and provide theoretical guidance for the subsequent selection of suitable nano-TiO_2_ for photocatalytic antibacterial appropriately, the mechanism of ROS production and utilization is summarized, and the factors affecting photocatalytic antibacterial efficiency are comprehensively discussed.

### 2.1. Photocatalysis and Antibacterial Properties

#### 2.1.1. Photocatalysis ROS Production Mechanisms

ROS production via an excellent photocatalytic process serves as the foundation for antibacterial activity. Nano-TiO_2_, a prevalent semiconductor catalyst with unique crystal configurations, can produce ROS under UV light irradiation. Through irradiation, high-energy photons were generated, and energy was transferred to the electrons in the valence band (VB), causing photogenerated electrons to transition from VB to the conduction band (CB) and leaving holes in the VB at the same time, forming the separation of electrons and holes (Figure 1). Photogenerated carriers are negatively charged and the left holes are positively charged, and they can, respectively, participate in redox reactions to produce ROS. This process may produce one or two types of ROS, of which type I ROS is produced mainly through a series of redox reactions caused by electron transfer, such as superoxide radical (O_2_**^·^**^−^), hydroxyl radical (·OH), singlet oxygen (^1^O_2_), and hydrogen peroxide (H_2_O_2_), and type II ROS is generated by energy transfer [4]. The production principles of different types of ROS are different. For example, the light carrier on the conduction band can reduce O_2_ to form O_2_**^·^**^−^, the hole in the valence band can oxidize water to form ·OH, and ^1^O_2_ can be generated in the transition and excited state through TiO_2_ energy transfer to O_2_. ROS produced directly by the photocatalysis of TiO_2_ can interconvert under certain conditions. For example, O_2_**^·^**^−^ and water can further react to form H_2_O_2_, and when the oxidation continues, O_2_ will be further oxidized to form ^1^O_2_.

#### 2.1.2. Antibacterial Mechanisms

The bactericidal efficacy of nano-TiO_2_ is predominantly exerted via the generation and interaction with ROS. ROS can disrupt the integrity of bacteria cell membranes, leading to the leakage of the cell matrix, DNA damage, and affecting the ROS-mediated redox (Figure 1). The exploration and discovery of the bactericidal effects of nano-TiO_2_ can be summarized as follows. Researchers found that the surface of *E. coli* cells became irregular after exposure to nano-TiO_2_, indicating that the cell membrane was disrupted, potentially leading to the leakage of the internal cellular components due to altered cell membrane permeability [12]. To further confirm the alteration of cell membrane permeability caused by disruption of bacterial integrity, Ranjan and Ramalingam used a 1-*N*-phenenaphthaline fluorescent probe (unable to enter the intact cell membrane of Gram-negative bacteria) to detect the cell membrane permeability of E. coli. They found that the *E. coli* suspension treated with nano-TiO_2_ showed a stronger light absorption value, indicating that the cell membrane was destroyed, which fully confirms that the induced disruption of bacterial cell wall integrity is the main mechanism of antibacterial activity [13]. ROS with strong redox properties can react with polyunsaturated phospholipids of bacterial cell walls, causing cell membrane puncture [14]. With the extension of light time, electrons and holes produced by nano-TiO_2_ involved in redox reaction are gradually enriched, and the degree of cell membrane destruction showed a dose-dependent effect [13]. Moreover, Ezati et al. suggested that TiO_2_ nanoparticles accumulate on the surface of bacterial cell walls and produce ROS to inhibit DNA replication, leading to cell rupture [15].

However, the destruction of cell integrity is only the beginning of cell death. Once bound to the bacterial cell wall, ROS were more easily able to damage and enter the bacteria, where they reacted with bacterial enzymes, DNA, and proteins in a diffusion-controlled manner, leading to enhanced antibacterial effects. Zhang et al. demonstrated, by flow cytometry, that disruption of cell wall permeability induced a sublethal state and that the sublethal state of *E. coli* O157:H7 was associated with increased intracellular ROS levels [16]. ROS can cause the proton-hydrodynamic potential (PMF) to collapse, which limits the energy requirements for the transfer of material inside and outside the cell membrane and affects the synthesis of ATP enzymes. Alternatively, ROS directly attack bacterial endogenous enzymes such as ATP enzymes, reducing their activity and affecting phosphate transfer, energy metabolism, and substance transport. At the same time, the accumulation of ROS leads to the destruction of the bacterial internal antioxidant defense system, and strong oxidative stress damage, making the bacteria unable to decompose and eliminate excess H_2_O_2_ and peroxide, and, finally, killing all bacteria after the resulting ROS attack [17], which is an important cause of bacterial death [16].

### 2.2. Factors Affecting Photocatalytic Antimicrobial Properties

**Figure 2:**
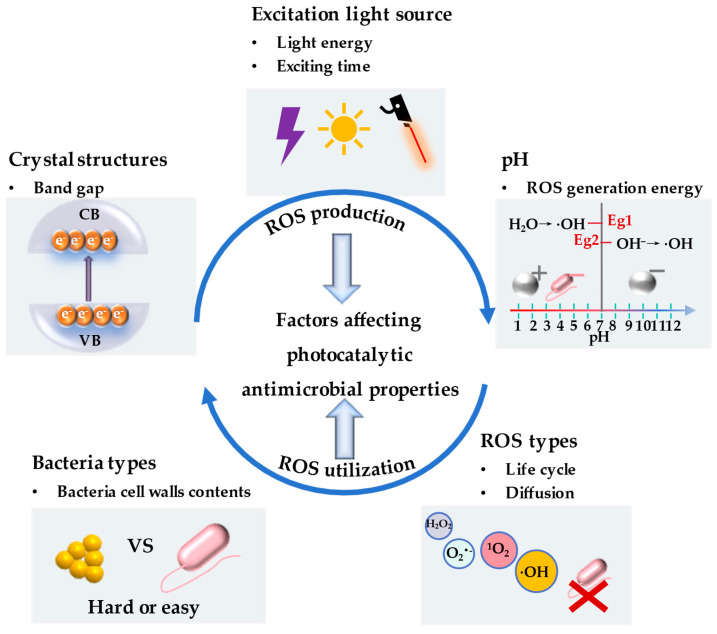
Factors affecting photocatalytic antibacterial properties of nano−TiO_2_.

The photocatalytic and antibacterial activity of nano-TiO_2_ is significantly influenced by the generation and utilization of ROS under the redox reaction triggered. The efficacy of ROS production and utilization is subject to various factors, including crystal configurations, light sources, environmental conditions, etc. This section delves into the impact of crystal structure, excitation light sources, pH, ROS species, and bacteria types on the photocatalytic activity of TiO_2_ (Figure 2). It provides a theoretical foundation for its application in post-textual photocatalytic antibacterial activity and offers guidance for further research in photocatalytic theory.

#### 2.2.1. Crystal Structure

The photocatalytic behavior of TiO_2_ is highly dependent on its crystal structure. TiO_2_ exists in three main crystal forms: anatase, rutile, and brookite. In the TiO_2_ crystal form, Ti^4+^ ions are surrounded by oxidized octahedral ions. In the anatase phase, each octahedral ion trades with eight adjacent octahedra (four oxygen atoms at the edge, four oxygen atoms share one corner), while, in the rutile structure, each octahedron is connected to ten adjacent octahedra (two sides and eight corners are shared) [8]. Among these, anatase is recognized for its superior photocatalytic performance due to its ability to absorb a wider range of the solar spectrum and its higher surface area, which facilitates more efficient charge carrier separation [18]. The synergistic effect of anatase and rutile in the P25 configuration, with approximately 75% anatase and 25% rutile, results in higher photocatalytic activity than either phase alone. However, the heterogeneous mixing of these configurations is not simply additive; the activity increases with a higher anatase content [19]. Moreover, the photocatalytic effects of single crystal surfaces with different crystal structures differ. Gunnemann et al. found that the surface activity of anatase (101) was higher than that of rutile (100), while the surface activity of brookite was the lowest. They also found that the reorganization of photogenerated charge carriers plays an important role in their surface chemical activity by testing photon production efficiency [20]. In addition, the depth of charge carriers caused by crystal structure also affects the consumption and recombination of photogenerated carriers. The presence of a too-deep electron trap causes the electrons to be unable to reach the surface of the catalyst to participate in the redox reaction, resulting in the lowest carrier generation rate and the highest carrier composite rate among all the tested materials, and the photocatalytic effect is poor [18].

#### 2.2.2. ROS Species

The types of ROS produced during photocatalytic reactions significantly influence the antibacterial activity of nano-TiO_2_. The generation of ROS, such as superoxide radicals (O_2_**^·^**^−^), hydroxyl radicals (·OH), and singlet oxygen (^1^O_2_), is critical in photocatalytic oxidation processes [21]. Different ROS species have distinct chemical properties and biological effects. These ROS species participate in different reaction pathways that affect the activity and selectivity of the reaction. For instance, among free radical ROS, the half-life of O_2_**^·^**⁻ is considered relatively long, ranging from a few milliseconds to several seconds in aqueous solutions. Its diffusion coefficient in water is 1.5 × 10^5^ cm^2^/s, and it is generally believed to exhibit low reactivity in water due to the fact that it is highly solvated by water molecules. Luo et al. found its redox potential in different solvents and its reduced reduction in water compared with organic solvents, which further confirmed that the solvent environment affects the reactivity of O_2_**^·^**^−^. ·OH is the most active radical with very low specificity for diffusion control reactions with neighboring molecules [22], with a short half-life on the nanosecond scale [23]. The life cycle of singlet oxygen is about 3 μs, and the penetration depth is from micron to millimeter [24], but the annihilation rate is fast, 100 ns. Moreover, the average diffusion distance of ROS in water and biofilms is 200 nm and 400 nm, respectively. Therefore, ROS are easily intercepted and quenched in a nutrient-rich aqueous medium [16].

ROS are important antimicrobial agents during PDI. In order to relate cell death to ROS, Xu et al. determined the type of ROS generated in an attempt to construct the link between ROS and antibacterial activity, and found that, among the three ROS measured (·OH, O_2_**^·^**^−^, and H_2_O_2_), ·OH and O_2_**^·^**^−^ diffusion was the key to the death of *E. coli* [24]. Although microbial cells can develop resistance by upregulating defenses [25] to some types of ROS such as O_2_**^·^**^−^ and H_2_O_2_, they are unable to defend themselves against others such as ·OH and ^1^O_2_ [26]. On the basis of measuring the amount of ^1^O_2_ and O_2_**^·^**^−^ generation, Yan et al. used transcriptomics to study the expression of genes related to the food spoilage fungus *A. johnnii* XBB1 and found that the photocatalytic process of TiO_2_ significantly inhibited cell autoregulation and membrane wall system repair, downregulated the expression of spoilage-related genes, and increased the production of secondary metabolites [27].

#### 2.2.3. Bacteria Types

Nano-TiO_2_ exhibits varying degrees of antibacterial effects against different types of bacteria (Table 1), which is attributed to the distinct characteristics of bacterial cell walls. Research indicates that nano-TiO_2_ has shown better antibacterial effects against Gram-positive bacteria, such as *Listeria* and *Staphylococcus aureus*, than Gram-negative bacteria like *E. coli* [28]. This preference may be linked to the structural differences in bacterial cell walls, where Gram-positive bacteria possess a single layer of peptidoglycan and lower lipid content, allowing for the easier penetration of antibacterial components [29]. For instance, Li et al. reported that a low concentration of nano-TiO_2_ (0.4%) was more effective against *S. aureus* than a higher concentration (0.6%) on *E. coli* [7]. Similarly, Golipour et al. observed that low concentrations of TiO_2_ inhibited *S. aureus* but had no effect on *E. coli* [30]. Bian et al. found that the same dose of TiO_2_ could inhibit 100% of *S. aureus*, while the inhibition rate for *E. coli* was only 70% [31].

However, some studies have reported that nano-TiO_2_ had a better antibacterial effect against *E. coli* than *S. aureus* [32]. This could be due to the thinner peptidoglycan layer of Gram-negative bacteria, which may allow for the easier absorption of free radicals [28]. Additionally, polar hydrophilic TiO_2_ particles and active substances are more likely to pass through the negatively charged lipopolysaccharide outer membrane of *P. aeruginosa* rather than the thick peptidoglycan cell wall of *S. aureus* [33].

**Table 1:** Antibacterial capacities of nano-TiO_2_.

Microorganism	TiO_2_	Irradiation Light	Evaluation of Antimicrobial Potential Main Outcome	Refs.
*Salmonella* *Escherichia coli* *Staphylococcus aureus*	6.3–11.1 nm	30 W UV light (λ = 254 nm, intensity 0.5 mW/cm^2^) for 60 min	The sizes of inhibition zones: *Staphylococcus aureus* 24.02 mm (0.4% TiO_2_) and *Escherichia coli* 24.02 mm (0.6% TiO_2_). The sizes of inhibition zones: *Salmonella* 21.34 mm (0.8% TiO_2_).	[7]
*Listeria monocytogenes* *Escherichia coli*	49–62 nm	white LED, wavelength with a maximum intensity of around 450 nm (blue region) and 530–620 nm (yellow region)	The number of *Listeria monocytogenes* and *Escherichia coli* decreased by 6 and 4 Log CFU/mL compared with the control group.	[15]
*A. johnnii* XBB1	10–25 nm, anatase	UV radiation (wavelength = 365 nm)	The number of bacteria and enzyme activity in the photocatalytic treatment group decreased more significantly than the control group and the dark treatment group.	[27]
*Escherichia coli* *L. monocytogenes*	/	/	The sizes of inhibition zones: *Escherichia coli* 3.5 cm and *L. monocytogenes* 4.5 cm.	[28]
*Staphylococcus aureus* *Escherichia coli*	25 nm, anatase	300 W xenon lamp, one solar light simulation, 1 h	Inhibition rate: 37.9% and 51.1% against *S. aureus* and *E. coli* for pure TiO_2_, and more than 90.0% for both *S. aureus* and *E. coli* for TA-TiO_2_.	[34]
*Streptococcus mutans* *Escherichia coli*	Zn-TiO_2_/reduced graphene oxide (HTGZ)	visible light at 300 mW/cm^−2^, 15 min	Inhibition rate: *Streptococcus mutans* 98.25%; stronger antibacterial effects against *S. mutans* than against *E. coli.*	[29]
*Staphylococcus aureus* *Escherichia coli*	/	/	Low content of nanoparticles only has an impact on *Staphylococcus aureus*.	[30]
*Staphylococcus aureus* *Escherichia coli*	CoPcTs-P25	250 W xenon lamp 15 kW/m^2^ at 400–800 nm, visible light irradiation	Inhibition rate: *Staphylococcus aureus* 100%; *Escherichia coli* 70%.	[31]
*Escherichia coli* *Staphylococcus aureus* *Candida albicans*	50–80 nm	visible light (20 W daylight lamp)	Inhibition rate: better antibacterial activity for *Escherichia coli* than other bacteria.	[32]
*Staphylococcus aureus* *Pseudomonas aeruginosa*	/	/	Inhibition rate: higher inhibition rate for *Staphylococcus aureus.*	[35]
*Staphylococcus aureus* *Escherichia coli*	P25	white LED	*Escherichia coli* 4.2 mm *Staphylococcus aureus* 5.6 mm Control: 1.0 and 1.2 mm	[36]
*Pseudomonas aeruginosa* *Staphylococcus aureus*	13–47 nm	/	The sizes of inhibition zones: *Pseudomonas aeruginosa* 19 mm and *Staphylococcus aureus* 16 mm.	[33]

#### 2.2.4. Excitation Light Source

To excite the electros transition and ROS production of nano-TiO_2_, energy input greater than or equal to the forbidden band width of nano-TiO_2_ is needed. Selecting a suitable excitation light source according to the band gap width is important to enhance the photocatalytic properties. There are three main polymorphs of nano-TiO_2_: anatase, rutile, and brookite, which differ in the band gap. Previous studies showed that anatase is 3.2 eV [37], rutile is (3.0 eV) [38], and brookite is (3.2 eV) [39]. All of them have large band gaps. In nature, ultraviolet light energy can reach 3.4 eV (higher than the large band gap of nano-TiO_2_), which means TiO_2_ can only be excited under UV light, and visible light does not work. However, there are many limitations to using UV light in practical food applications, such as being harmful to the human body and requiring additional equipment, high cost, and destructive effects on certain materials. For food packaging applications, the utilization of visible light is favored because it reduces safety management costs and energy consumption, as well as improving convenience [40]. Thus, the visible light excitation photocatalytic antibacterial activity of nano-TiO_2_ has attracted wide attention, and important progress has been made in recent years. This article does not elaborate on this too much.

#### 2.2.5. pH Levels

The pH has a significant impact on photocatalysis in practical applications. In alkaline conditions, ·OH is one of the main ROS that works. Compared to H_2_O (H_2_O/·OH = 2.38 eV), OH^−^ was more easily converted into ·OH (OH^−^/·OH = 1.99 eV). Wang et al. suggested that ·OH plays an important role in degrading MB [41], for which the pH of reaction systems has an impact on the ROS types generated by nano-TiO_2_. Moreover, the pH can influence the stability and dispersion of nano-TiO_2_ nanoparticles in the polymer matrix. Nanomaterials containing nano-TiO_2_ adsorbed more OH^−^, causing its surface charge to become negative, while, under an acidic environment, H^+^ was adsorbed, and the surface charge became positive, which, in turn, affects the contact between the nanoparticles and the bacteria, and, thus, the antibacterial performance [42]. Therefore, by adjusting the pH, ROS can be generated selectively to inactivate the target.

## 3. Application of Nano-TiO_2_ in Food Antimicrobial Packaging Materials

### 3.1. Current and Emerging Strategies to Prepare Nano-TiO_2_-Based Antimicrobial Packaging

**Figure 3:**
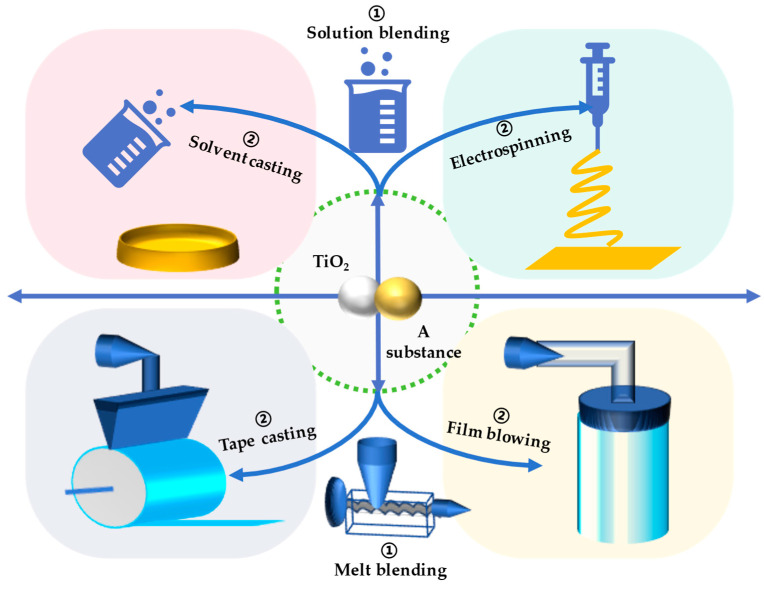
Preparation methods of nano-TiO_2_-based food packaging films.

Nano-TiO_2_, with its excellent photocatalytic antibacterial properties, can be incorporated into food packaging films to enhance their functionality, particularly in terms of antimicrobial activity. The preparation of nano-TiO_2_-based food packaging films involves a variety of methods (e.g., solution casting, melt blending, extrusion, blowing, and electrospinning), which will be discussed briefly in the following sections (Figure 3, Table 2).

#### 3.1.1. Solvent Casting

Solvent casting is a prevalent method for producing food packaging in labs due to its simplicity, low equipment requirements, and efficiency. The process involves the preparation of the polymer solution, incorporation of nano-TiO_2_, de-gassing, casting, and drying (Figure 3). Well-dispersed and de-gassed nano-TiO_2_ is crucial for obtaining a smooth and defect-free film surface, which is generally achieved through ultrasonic treatment. Films with lower TiO_2_ content exhibited good dispersion, but, as the content increased, surface unevenness and aggregation occurred, suggesting that high TiO_2_ concentrations (0.8% and 1.0%) could not be adequately dispersed by sonication alone. This finding aligned with antibacterial tests, which showed a decrease in antibacterial properties as TiO_2_ content rose to 0.8% in PLA [7]. Similar observations were made by other researchers, indicating a correlation between TiO_2_ dispersion and antibacterial activity. To improve dispersion, some studies modified nano-TiO_2_ properties through doping or additives, such as copper doping or the addition of nisin, which enhanced particle dispersion in chitosan films [27]. These modifications help to better integrate nano-TiO_2_ into the film, preventing particle migration during application. Therefore, optimizing nano-TiO_2_ properties or content is important for achieving superior packaging film properties, with a focus on improving particle dispersion.

#### 3.1.2. Melt Blending

Melt blending is a more adaptable technique specifically for thermoplastic polymers. Because of this, this method is found to be environmentally friendly, cost-effective, and best for mass production [65]. The polymer and nano-TiO_2_ are mixed at temperatures above the melting point of the polymer, ensuring a thorough blend without the need for solvents. Beginning with the melt blending, there are several methods forming homogeneous films (e.g., blowing [43], tap casting [28], hot pressing, and co-extrusion [44]) (Figure 3). This technique is advantageous for its simplicity and the ability to create films with good barrier properties. This method is particularly beneficial as it allows for better control over the dispersion of nano-TiO_2_, which is crucial for maintaining the film’s integrity and performance. However, it also poses challenges, as some polymer matrices can be unstable and prone to degradation at high temperatures, which may limit their applicability.

#### 3.1.3. Electrospinning

Electrospinning technology is a cutting-edge process for preparing nanofiber membranes, renowned for its simplicity and versatility. This technique involves dispersing nano-TiO_2_ within a polymer matrix, which is then dissolved in organic solvents such as DMSO, dichloromethane, or chloroform. The solution is electrified and ejected through a thin nozzle under high voltage, forming a fiber that accumulates into a nanofiber membrane as the solvent evaporates [66].

After doping with nano-TiO_2_, the resulting nanofibers exhibited a high specific surface area and porosity, with the nanoparticles being evenly distributed across the fiber surface. Recent studies showed that this uniform distribution was particularly evident at nano-TiO_2_ concentrations of 1.25 wt.% or less. Of note, the photocatalytic reaction of TiO_2_ was achieved by direct contact between the free radicals and test bacteria. TiO_2_ nanoparticles easily aggregated in the polymer matrix and reduced the photocatalytic reaction when increasing its content. Research by Bian et al. has demonstrated that CoPcTs-P25 nanoparticles integrated into the nanofibers displayed significant antibacterial activity under visible light, with complete bacterial inhibition against *S. aureus* and *E. coli* within specific timeframes. However, when the TiO_2_ concentration reached 1.50 wt.%, the fiber morphology was significantly altered due to the loading of nano-TiO_2_. The antibacterial efficacy was observed to be the highest at lower TiO_2_ concentrations, aligning with SEM image results [31]. Feng et al. suggested that the inhibition effect of nanofibers is more efficient than that of films, highlighting the superior performance of electrospun nanofibers in certain applications [67]. Electrospinning is a sophisticated method for producing nanofiber membranes with tailored properties for various applications. While challenges remain in terms of scalability and cost, ongoing research continues to address these issues and enhance the technology’s efficacy.

Overall, the characteristics of food packaging films produced through different manufacturing processes exhibit discernible differences. To obtain packaging films with specific desired properties, it is crucial to choose the right processing techniques by taking into account both the characteristics of the polymers and the practical aspects of the company’s production capabilities.

### 3.2. A Guide to Different Approaches to Evaluate the Antimicrobial Properties

To ensure the stability of the antimicrobial activity throughout the packaging process, storage, and until the food product is consumed, the antimicrobial property evaluation of nano-TiO_2_ food packaging materials must be considered [68]. In published works on nano-TiO_2_-based antimicrobial packaging, the authors have used different in vitro methodologies to investigate the antimicrobial performance of nano-TiO_2_-based antimicrobial packaging, both in culture media and food models. To stimulate real-world scenarios of film antimicrobial applications, films are often required to be co-cultured with bacteria in the broth or on the agar, film, or food model (Table 2) before or after light treatment. According to the different antibacterial properties of the antibacterial determination test method, the antibacterial properties can be visualized by comparing the size of the inhibition zone or the total number of bacterial colonies or transformation into the antibacterial rate.

#### 3.2.1. Broth Co-Cultured Method

The broth co-culture method is a widely recognized technique for assessing the antibacterial properties of films. In this method, films are typically immersed in a broth medium inoculated with bacteria and then treated with light while being incubated under specific conditions [15]. The antibacterial activity is determined by viable cell counts, which can be measured on agar media, or by monitoring the optical density through spectrophotometry. Additionally, the reduction in bacterial growth can be estimated using growth inhibition equations, such as Equation (1), mentioned in various studies. It is important to note that, in these experiments, both sides of the nano-TiO_2_-based films often come into complete contact with bacteria, which contrasts with real food preservation scenarios where, typically, only one side of the film is in contact with the food. This difference is crucial when interpreting the results of antibacterial tests:

(1) $$Growth\;inhibition\;\left(\%\right)=\frac{A-B\;}{A}\times 100$$

where *A* and *B* are average viable cells and colony-forming units (CFU) in the control sample and treatment at a specific time, respectively [55].

#### 3.2.2. Agar Co-Cultured Method

The agar-based diffusion plate method, a popular and cost-effective technique for testing antibacterial activity, offers simplicity and flexibility. This method begins with the preparation of agar plates coated with a bacterial suspension. Films of uniform size, typically 6 mm in diameter or larger, are then placed on these plates. The films are subjected to light, activating the nano-TiO_2_ and prompting the generation of ROS that inhibit bacterial growth. The presence of inhibition zones around the film areas indicates the antibacterial effect, with the size of these zones reflecting the film’s antibacterial potency.

The agar-based method more closely simulates the conditions of wrapped food compared to broth co-culture methods, making it particularly suitable for antibacterial testing in food packaging applications. It allows for a direct visualization of the antibacterial effect, which is a significant advantage over broth methods that require additional steps to quantify bacterial growth inhibition.

#### 3.2.3. Film Co-Cultured Method

Typically, the film co-cultured method is used for evaluating in vitro antibacterial activity of nanomaterials. Unlike films actively in contact with bacteria, this method spreads bacteria suspension (usually 100 μL) on the surface of the film, which were then placed in a purified horizontal clean bench at proper ambient temperature and relative humidity under light irradiation [32]. After treatment, bacteria were washed from films using sterile water and spread on an agar plate. The colony-counting method can be used to evaluate the antibacterial activity of films. However, this procedure is more complicated than previous methods. We suggest that this method is more suitable for testing the antiadhesive properties of nano-TiO_2_-based film against bacteria outside the packaging than antibacterial activity against bacteria on food surfaces.

#### 3.2.4. Food Model Co-Cultured Method

The food model co-cultured method is usually used in food storage experiments. Experimenting on the food matrix directly, this method fabricated the same environment of packaging to preserve food, that is, using film to wrap food and store it for a specific time, and testing the viable cell counts on the food surface to determine the antibacterial activity of nano-TiO_2_-based packaging. In addition, some researchers also use the quality of food to determine the antibacterial activity indirectly. However, its experiment is longer than other methods, which requires more money and time than the in vitro antibacterial method. As a result, it is suitable for further verification after the film has a certain antibacterial effect or before it is applied in food preservation.

In conclusion, the detection of antibacterial activity of the film is very important for the future application of the film, and standard evaluation methods should be established to evaluate the antibacterial properties of the film. In addition, the standard methods for the antibacterial performance assessment of spoilage bacteria in many published studies need further improvement.

### 3.3. Application in Food Preservation

The photodynamic antibacterial strategy, which produces ROS upon irradiation, not only offers a green and efficient approach with a broad spectrum of stable antibacterial activity, but also paves the way for innovative antibacterial packaging solutions. Particularly, nano-TiO_2_, with its exceptional photodynamic properties, has garnered significant research interest and is increasingly being tapped for its potential in food preservation. The following outlines its progress in food antibacterial packaging applications in recent years (Table 2).

Fresh foods like pork, beef, and fish are prone to microbial contamination in the production and processing chain, leading to quality deterioration. The initial number of microorganisms on the food surface has a significant effect on the rate of food deterioration, which can be solved by adding nano-TiO_2_ in food packaging [69]. Marcous et al. mixed composite membranes with LDPE and found that the composite membrane reduced the number of bacteria by 90.3% [5]. Siripatrawan and Kaewklin tested the antibacterial activity of a nano-containing chitosan membrane against four bacteria and two fungi, and found that all microorganisms tested were inhibited and had a stronger inhibitory effect on bacteria, which could be used in the post-harvest packaging of fruits and vegetables in the future [56]. Goñi-Ciaurriz and LVélaz I determined the activity of bacteria after 3 and 18 h of UV irradiation, and the results showed that TiO_2_-containing packaging effectively inhibited bacterial growth with the lowest bacterial activity (39%) [70].

Moreover, nano-TiO_2_-based packaging film shows good antibacterial activity in the practical application of fresh food, especially in extending the shelf life of food. It was found that the microbial contamination of directly packaged beef terminals could be reduced by 51.8% [5]. After 15 min of UV activation, total colonies and *E. coli* numbers of pork packaged in non-woven fabric containing nano-TiO_2_ decreased by 1.35 and 1.39 log, respectively, whereas the total number of colonies and *E. coli* numbers of the blank control group decreased by only 1.07 and 0.88 log after UV irradiation for 60 min, respectively. These results showed that nano-TiO_2_ played an important antibacterial role in reducing the initial number of microorganisms in pork [6]. Li et al. conducted a 6-day storage test and determined the total number of pork colonies. The total colonies of PLA/nano-TiO_2_-film-packaged pork compared to the control group changed very slowly, and extended the shelf life of pork from 2 days to 3 days at 25 °C and from 5 days to 6 days at 4 °C [7], which further confirmed that nano-TiO_2_-based film can reduce the initial microbial quantity, maintain food quality, and extend the shelf life of fresh food. In addition, nano-TiO_2_-based film can also be applied to fruits and vegetables. Foodborne pathogenic bacteria and fungi are the common microorganisms in fruits and vegetables. Du et al. inoculated root mold into fresh strawberries and tomatoes to verify the preservation effect of antibacterial compound film, which confirmed that the UV-film-activated antibacterial activity could maximize the good appearance of tomatoes [55].

Altogether, titanium dioxide-based food packaging is widely applied and has proven to be effective in food preservation, including fresh meats such as beef, pork, and fish and fruits like strawberries, bananas, and kiwis, as well as cooked foods such as bread. Despite the promising outcomes from numerous laboratory-based experiments, there remains a significant gap to bridge before these can be effectively transitioned into industrial-scale production. The challenge of cost reduction and the complexities of scaling up from pilot-scale studies to full-scale industrial applications are substantial hurdles that require targeted research and development efforts.

### 3.4. Migration of Nano-TiO_2_ from Packaging into Food

Recently, the European Union (EU) banned TiO_2_ as a food additive due to its potential genotoxicity by ingestion, but it is still allowed in food packaging. However, migration studies have shown that nano-TiO_2_ can leach into foodstuffs [71,72,73]. Consuming food that has come into contact with such packaging raises concerns regarding possible health hazards [74]. To enhance our understanding of the safety profile of nano-TiO_2_-based food packaging, further research is imperative.

The migration of chemical substances from packaging into food is affected by many parameters such as food properties, types of polymer matrixes, TiO_2_ concentration, temperature, and food storage time. Packaging food properties affect the migration of nano-TiO_2_, especially the pH of food substrate. The migration levels of TiO_2_ were higher in acidic settings than in other simulants [72]. Migrants tend to choose paths with the least diffusion resistance in the polymer chain, usually avoiding the positions occupied by nanoparticles, resulting in longer paths [75]. The interaction of PLA nanocomposite films with ethanol solution led to the degradation of polymer chains in amorphous regions, increasing the crystallinity and reducing the amorphous content. This process may slow down the release of nano-TiO_2_ due to secondary crystallization. Over time, the films’ surface became rougher, enhancing the interaction with ethanol and potentially increasing the migration of nanoparticles [71]. Forooghi et al. found that the better dispersion of TiO_2_ particles in amorphous starch hindered the release of Ti from the polymer chains, giving reduced migration levels in all simulants [76]. Ning et al. fabricated multifunctional food packaging composite films using 25 nm TiO_2_, exopolysaccharide (EPS), and potato starch (PS) through the casting method. Results showed that the migration of Ti element was 4.35 ± 0.05 mg/kg on the 10th day during chilled meat storage, which was lower than the maximum migration of 10 mg/kg allowed by the European Food Safety Authority (EFSA) [48]. The migration levels reported in various studies exhibit discrepancies due to differences in experimental methodologies. Hatice et al. observed that the migration of Ti nanoparticles into minced meat was 12.1 μg/kg in samples exposed to 3% acetic acid at 100 °C for 8 h and 48.15 ppm in the 3% TiO_2_ containing samples stored at 1 °C and 7 days [73]. They also found that the migration increased as the concentration and time increased from the TiO_2_-containing films, and the effect of temperature on the migration was insignificant. In Isabel et al.’s study, the Ti migration during storage is expected to be negligible under standard use of the products [77].

## 4. Conclusions and Future Perspectives

In conclusion, nano-TiO_2_ has excellent photodynamic sterilization activity and has been extensively studied in antibacterial food packaging materials. Its integration into packaging materials enhances antimicrobial properties, ensuring food safety and extending shelf life. The efficacy of nano-TiO_2_ is contingent upon the generation and utilization of ROS, a process influenced by various factors, including the crystal structure of nano-TiO_2_, ROS types, bacterial types, light source, and pH levels. In fact, in the future production and application of nano-TiO_2_ antibacterial food packaging materials, antibacterial activity is the focus of enterprises. In addition, the application of TiO_2_ in food packaging materials also faces challenges.

(1) Mechanism understanding: In-depth research on the chemical reaction mechanisms involving ROS and antimicrobial mechanisms is essential for the design and modification of efficient nano-TiO_2_-based photocatalytic antimicrobials. This includes exploring the potential against typical food spoilage bacteria of nano-TiO_2_ to broaden its application in food preservation.
(2) Material dispersion and polymer properties: Achieving the excellent dispersibility of nano-TiO_2_ within the polymer matrix and ensuring the polymer’s mechanical properties and sustainability are key to realizing the practical application of nano-TiO_2_-based food packaging. Dispersion within the polymer matrix is also one of the essential factors involved in the antibacterial efficiency of food packaging. Metallic oxides often tend to self-aggregate in a polymer matrix, significantly impacting their photocatalytic ability. Hence, it is important to improve the dispersion degree of nano-TiO_2_ to increase the antibacterial contact area.
(3) Safety and efficacy evaluation: Different detection methods may yield disparate results, which complicates the accurate assessment of packaging performance. There is an urgent need for the standardization and unification of evaluation methods for the migration of titanium dioxide and the antibacterial performance of nano titanium dioxide-based food packaging. Standardized and unified evaluation methods are essential for ensuring the safety, efficacy, and market acceptance of food packaging materials. The adoption of standardized testing methods can enhance the comparability and reliability of data, thereby facilitating the industrial application of nano titanium dioxide-based food packaging materials.
(4) Visible-light-catalyzing antibacterial nano-TiO_2_ development: Being excited by UV light is one of the significant challenges scientists confront. UV light, which has higher energy than visible light, poses a huge threat to food quality, especially for protein-rich foods, such as meat and fish products. Therefore, it is not suitable for the preservation of fresh food. To better use nano-TiO_2_ to kill bacteria without oxidant protein, it is important to enhance its antibacterial properties under visible light.

## Figures and Tables

**Table 2:** Application of nano-TiO_2_ in food antimicrobial packaging materials.

Nanofillers	Polymer Matrix	Film Processing	Antimicrobial Co-Culture	Evaluation of Antibacterial Properties	Food Preservation Applications: Main Outcome	Refs.
TiO_2_	nonwoven fabric	coating	food model co-cultured	colony counting	Pork: the number of TVC and *E. coli* achieved 1.35 and 1.39 log reductions, which were higher than the control group (1.07 and 0.88 log).	[6]
TiO_2_	polylactic acid (PLA)	solvent casting	agar co-cultured	inhibition zones	Pork: the decrease in protein decomposition inhibited the growth and reproduction of microorganisms and resulted in slower pH change.	[7]
Cu–TiO_2_	carboxymethyl cellulose	solvent casting	broth co-cultured	colony counting	Banana: extending its post-harvest shelf life to 14 days.	[15]
TiO_2_–nisin	chitosan	solvent casting	food model co-cultured	colony counting	Chilled pork: prolonging the fresh-keeping period of chilled pork at 4 °C to 20 days.	[27]
TiO_2_	starch/polyvinyl alcohol (PVA)	solvent casting	broth co-cultured	inhibition zones	/	[28]
CoPcTs-P25	PVA	electrospinning	broth co-cultured	colony counting inhibition rate	Strawberries: extending its shelf life from 3 to 7 days.	[31]
TiO_2_	chitosan	solvent casting	film co-cultured	colony counting	Red grape: extending its shelf life from 15 to 22 days.	[32]
TiO_2_	LDPE	melt blending	agar co-cultured	inhibition zones	/	[33]
poly(tannic acid)-TiO_2_	chitosan	solvent casting	broth co-cultured	colony counting inhibition rate	Grapes and kiwifruit: maintaining the best freshness for kiwifruit and reducing water loss for grapes.	[34]
TiO_2_/curcumin	pectin/gelatine	solvent casting	agar co-cultured	inhibition zones	Salmon fillet: extending its shelf life up to 12 days for 5 wt% TiO_2_.	[35]
CMP/TiO_2_	ethyl cellulose	solvent casting	agar co-cultured	inhibition zones	/	[36]
TiO_2_	LDPE	melt blending and blowing	food model co-cultured	colony counting	Tomatoes: the treatment group had the least fungus (2.5 log CFU/g), while the control group had the most (9.4 log CFU/g).	[43]
TiO_2_	PLA	solvent coating	agar co-cultured	inhibition zones	/	[44]
ZnO-TiO_2_	polyvinyl Butyral (PVB)	electrospinning	broth co-cultured	colony counting inhibition rate	Tomatoes and strawberries: delaying the ripening of tomatoes and maintaining the most hardness of strawberries after 10 days.	[45]
TiO_2_	PLA	solvent casting	agar co-cultured	inhibition zones	/	[46]
TiO_2_	PLA	solvent casting	broth co-cultured	colony counting	/	[47]
TiO_2_	exopolysaccharide and potato starch	solvent casting	agar co-cultured	inhibition zones	Fresh pork (tenderloin): TVC value of chilled pork still lower than 6 log CFU/g on the 10th day.	[48]
TiO_2_	carboxylated cellulose nanofibers	solvent casting	film co-cultured	colony counting	Strawberries and banana: delaying the transpiration and respiration of strawberries and browning and senescence of banana.	[49]
GO/TiO_2_	bacterial cellulose	immersing	agar co-cultured	colony counting	/	[50]
Cu-TiO_2_	PLA	solvent casting	agar co-cultured	inhibition zones	/	[51]
TiO_2_	chitosan	solvent coating	agar co-cultured	inhibitory zones	/	[52]
Ag + Cu/TiO_2_	LDPE	melt blending and blowing	agar co-cultured	inhibition zones	Tilapia fish: without lose its nutritional properties in this nanomaterial packaging.	[53]
Ag-graphene-TiO_2_	PLA	melt blending and blowing	broth co-cultured	inhibition rate	Curd cheese: extending its shelf life from 10 to 17 days.	[54]
THY@β-CD/TiO_2_	fish gelatine	solvent casting	broth co-cultured	colony counting	Strawberries and tomatoes: much less water loss to strawberries after 7 days and greener and more unripe to tomatoes compared with the control group.	[55]
TiO_2_	chitosan	solvent casting	broth co-cultured	colony counting	/	[56]
TiO_2_	PLA/chitosan	solvent casting	food model co-cultured	colony counting	Rye toast: reducing the mold count in rye bread.	[57]
lignin-TiO_2_	poly(butylene adipate-co-terephthalate	melting and hot-pressing	agar co-cultured	inhibitory zones	Strawberries: keeping an attractive appearance and free from fungal growth after 10 days of storage.	[58]
TiO_2_	*Manilla tamarind*/chitosan	solvent casting	agar co-cultured	inhibitory zones	/	[59]
Ag@TiO_2_	PLA and PE	melting and extruding	broth co-cultured	colony counting	*Beluga sturgeon*: PE + 3% Ag@TiO_2_ showed the most inhibition against the growth of microorganisms (4.9 × 10^7^ CFU/mL); PLA and PE had the most colony count equal to 6.1 × 10^7^ CFU/mL and 5.5 × 10^7^ CFU/m.	[60]
TiO_2_	tomato seed mucilage and gelatine	solvent casting	agar co-cultured	inhibitory zones	The incorporation of 1% TiO_2_ proved to be efficacious in impeding the proliferation of bacteria, mold, and yeast.	[61]
TiO_2_-tannic acid	soybean protein isolated/cellulose nanofibers	solvent casting	food model co-cultured	colony counting	Carp fillets: extending its shelf life from 5 to 11 days.	[62]
vanadium (V)-doped TiO_2_	polypropylene food container	atomic layer deposition	food model co-cultured	colony counting	Raw cow’s milk: the growth of MAnFAM was (5.3 ± 0.03) × 10^5^ CFU/mL in the control (uncoated) containers and (1.0 ± 0.16) × 10^5^) CFU/mL in the coated containers after 9 days.	[63]
TiO_2_	glycerol monolaurate-gelatine-zein bilayer films	solvent casting	film co-cultured	colony counting	Maximum inactivation was observed for films containing GML in gelatine and both layers.	[64]
